# PROTACs are effective in addressing the platelet toxicity associated with BCL-X_L_ inhibitors

**DOI:** 10.37349/etat.2020.00017

**Published:** 2020-08-31

**Authors:** Peiyi Zhang, Xuan Zhang, Xingui Liu, Sajid Khan, Daohong Zhou, Guangrong Zheng

**Affiliations:** 1 Department of Medicinal Chemistry, College of Pharmacy, University of Florida, Gainesville, FL 32610, USA; 2 Department of Pharmacodynamics, College of Pharmacy, University of Florida, Gainesville, FL 32610, USA; University of Southampton, UK

**Keywords:** Apoptosis, BCL-X_L_, navitoclax, proteolysis targeting chimera, thrombocytopenia

## Abstract

BCL-X_L_ is an anti-apoptotic protein that plays an important role in tumorigenesis, metastasis, and intrinsic or therapy-induced cancer drug resistance. More recently, BCL-X_L_ has also been identified as a key survival factor in senescent cells. Accumulation of senescent cells has been indicated as a causal factor of aging and many age-related diseases and contributes to tumor relapse and metastasis. Thus, inhibition of BCL-X_L_ is an attractive strategy for the treatment of cancer and extension of healthspan. However, development of BCL-X_L_ inhibitors such as navitoclax for clinical use has been challenging because human platelets depend on BCL-X_L_ for survival. In this review, the authors discuss how BCL-X_L_-targeted proteolysis targeting chimeras (PROTACs) afford a novel approach to mitigate the on-target thrombocytopenia associated with BCL-X_L_ inhibition. The authors summarize the progress in the development of BCL-X_L_ PROTACs. The authors highlight the *in vitro* and *in vivo* data supporting that by hijacking the ubiquitin protein ligase (E3) that are poorly expressed in human platelets, BCL-X_L_ PROTACs can significantly improve the therapeutic window compared to conventional BCL-X_L_ inhibitors. These findings demonstrated the potentially broad utility of PROTAC technology to achieve tissue selectivity through recruiting differentially expressed E3 ligases and to reduce on-target toxicity.

## Introduction

**Figure 1.**
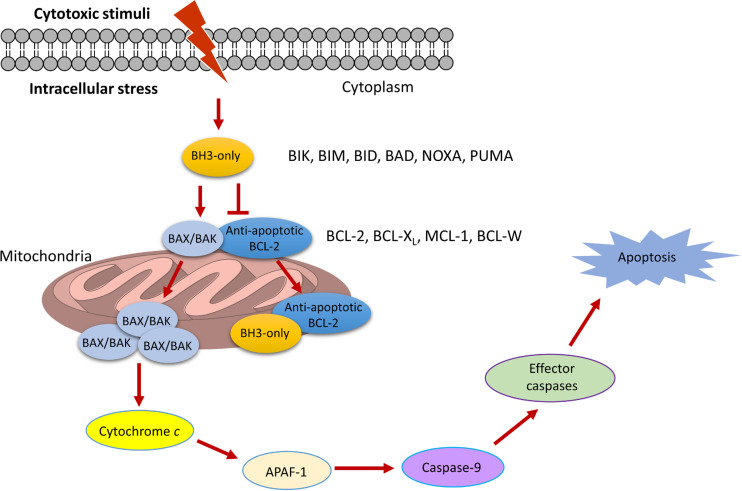
BCL-2 family proteins regulate mitochondria-mediated intrinsic apoptosis pathway

Apoptosis is a tightly regulated programmed cell death process that plays a vital role in controlling tissue homeostasis. Dysregulation of apoptotic pathways, thereby causes resistance to apoptosis, is a common feature of cancer cells and is also responsible for drug resistance triggered by apoptosis-inducing cancer therapies [1]. Therefore, sensitizing cancer cells to apoptosis is a promising therapeutic strategy for cancer. The B-cell lymphoma 2 (BCL-2) family proteins, consisting of pro- and anti-apoptotic members, are key regulators of the intrinsic apoptotic pathway. They control cell apoptosis by modulating the mitochondrial outer membrane permeabilization (MOMP) via protein-protein interactions (PPIs) between the proand anti-apoptotic proteins [2–4]. Anti-apoptotic BCL-2 proteins, including BCL-X_L_, MCL-1, BCL-W, and BFL-1/A1, inhibit MOMP and all have multiple BCL-2 homology (BH) domains. Depending on the structures and the functions they involve in apoptosis regulation, pro-apoptotic BCL-2 proteins can be divided into two subgroups, including BH3-only and multiple BH pore-forming proteins. BH3-only proteins, such as BIK, BIM, BID, BAD, BMF, HRK, NOXA, and PUMA, contain a single BCL-2 BH3 domain, while pore-forming BCL-2 proteins including BAX and BAK contain multiple BH domains. Anti-apoptotic BCL-2 proteins interact with BAX/BAK and prevent them from homo-oligomerization and subsequent pore formation in mitochondrial outer membrane. Upon the stimulation of apoptotic signals, the BH3-only proteins promote apoptosis by directly activating BAX and BAK and/or displacing them from the anti-apoptotic partners. Subsequently, BAK/BAX facilitates MOMP via homo-oligomerization and pore formation in the mitochondrial outer membrane, which causes the efflux of cytochrome *c* from mitochondria into the cytoplasm. Once released in the cytoplasm, cytochrome *c* binds with apoptotic protease activating factor-1 (APAF-1), which stimulates caspase 9 to activate the effector caspases and the eventual induction of apoptosis (Figure 1) [5].

**Figure 2.**
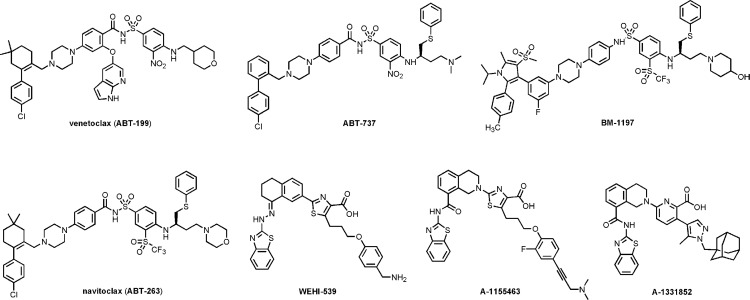
Structures of venetoclax and representative BCL-X_L_ selective (WEHI-539, A-1155463, and A-1331852) and BCL-X_L_/BCL-2 dual inhibitors (ABT-737, BM-1197, and navitoclax)

Anti-apoptotic BCL-2 family proteins are upregulated in many cancers and are associated with tumor initiation, progression, and resistance to cancer therapies [6–8]. Thus, inhibiting the PPI between anti- and pro-apoptotic BCL-2 proteins, thereby directly induces apoptosis in cancer cells, is an attractive cancer therapeutic strategy [9–11]. Many “BH3 mimetic” small-molecule inhibitors (SMIs) of the anti-apoptotic BCL-2 proteins, including BCL-2, BCL-X_L_, and MCL-1, have been developed [11–13], among which venetoclax (ABT-199, Figure 2) [14], a BCL-2 selective inhibitor, has been approved by the FDA for the treatment of refractory chronic lymphocytic leukemia (CLL) in 2016 [15].

Proteolysis targeting chimeras (PROTACs) are heterobifunctional molecules that act by recruiting the E3 ligase to degrade the protein of interest (POI) through the ubiquitin proteasome system. PROTAC technology is a paradigm-shifting approach for targeted protein degradation [16]. The last few years have witnessed rapid progress on this drug discovery platform. PROTAC degraders have been successfully developed for many clinically validated drug targets with two currently in clinical trials for cancer therapy [17]. Several PROTACs that target BCL-2 family proteins have been reported recently [18–21]. This review will summarize the progress on the development of BCL-X_L_-targeting PROTACs. We focus on demonstrating the rationale of utilizing PROTAC approach to overcome on-target toxicity. We will also discuss the potential limitations and future directions of this approach.

## BCL-X_L_ and BCL-X_L_ inhibitors

The therapeutic strategy of targeting anti-apoptotic BCL-2 proteins has been clinically validated by the FDA approval of venetoclax for the treatment of various hematologic malignancies including CLL and SLL as a single agent, and for AML in combination with low-intensity chemotherapy [22]. However, the complete remission rate (20%) of venetoclax-treated CLL patients is relatively low considering the overall response rate is 71–79% [23, 24]. This could be attributed to the upregulation of BCL-X_L_ by microenvironmental survival signals [25]. In addition, venetoclax has limited use in solid tumors because BCL-2 upregulation is mainly associated with the survival of hematologic malignancies and is unimportant for the survival of most of the solid tumors [26, 27]. Across the anti-apoptotic BCL-2 family members, BCL-X_L_ is the most frequently overexpressed protein in solid tumors and a subset of leukemia and lymphoma [28]. Tumor resistance to anticancer therapies has also been found to be positively correlated with BCL-X_L_ expression [29]. Besides being a cancer target, BCL-X_L_ is also essential for the survival of different senescent cells; therefore, BCL-X_L_ inhibitors have recently been identified as potent senolytics, i.e. molecules that can kill senescent cells while sparing normal cells [30–32]. As demonstrated in many studies, clearance of senescent cells by senolytics has the potential of treating many age-related diseases and cancer-therapy-induced short-/long-term adverse effects while inhibiting tumor relapse and metastasis [33]. Taken together, BCL-X_L_ is one of the most promising therapeutic targets for cancer as well as age-related diseases.

A number of BCL-X_L_ selective or BCL-X_L_/BCL-2 dual inhibitors have been developed over the past 15 years (Figure 2). ABT-737 was the first potent BCL-X_L_/BCL-2 dual inhibitor developed via fragment-based drug discovery by NMR approach [34, 35]. Navitoclax (ABT-263) [36] is an orally bioavailable analog of ABT-737 and has been in Phase II clinical trials for hematological malignancies and small cell lung cancer (SCLC). However, ABT-263 treatment leads to rapid and dose-dependent thrombocytopenia [37] because platelets depend on BCL-X_L_ to maintain their viability [38, 39]. Consistently, dose-limiting thrombocytopenia has also been observed in animals treated with other BCL-X_L_ inhibitors, such as ABT-737 [34], BM-1197 [40], and A-1155463 [41]. Thus, thrombocytopenia is an on-target toxicity of BCL-X_L_ inhibitors that cannot be solved by conventional medicinal chemistry.

Several strategies have been used to mitigate the on-target thrombocytopenia of BCL-X_L_ inhibitors. For example, ABT-263 has been combined with either chemotherapy or targeted therapies so that the platelet toxicity can be manageable due to the reduced clinically effective dose [42, 43]. However, the combination of ABT-263 with docetaxel exacerbated neutropenia in clinical trial, which is believed to be associated with its BCL-2 inhibitory effect [44]. In this case, BCL-X_L_ selective inhibitors such as WEHI-539 [45], A-1155463, and A-1331852 [46] (Figure 2) might be more suitable for combination therapy [44]. An alternative strategy is to design prodrugs to minimize drug exposure to human platelets. APG-1252, a phosphate prodrug derived from a BCL-X_L_/BCL-2 dual inhibitor [47], is currently under investigation in Phase I clinical trial (identifier: NCT0308031) [48]. AZD-0466, a dendrimer-conjugated dual BCL-2/BCL-X_L_ inhibitor, has also been shown to have an improved therapeutic index and is currently in phase I clinical trial (identifier: NCT04214093) [49]. Moreover, a BCL-X_L_-targeting antibody-drug conjugate (ADC) named ABBV-155 has also entered Phase I clinical trial (identifier: NCT03595059).

## PROTACs

**Figure 3.**
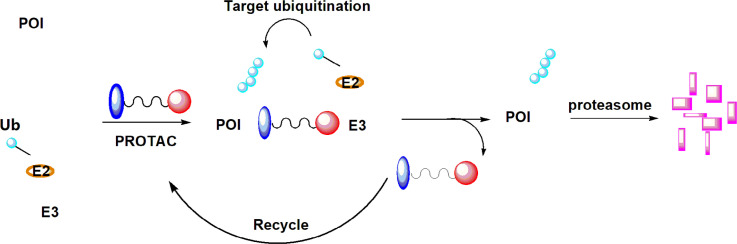
Mechanism of PROTAC-induced POI degradation

Since it was initially reported in a proof-of-concept study by Sakamoto et al. [50], in 2001, PROTAC technology has attracted tremendous interests from both industry and academia. PROTACs contain two distinct pharmacophores that are connected via a linker unit; one pharmacophore can bind to the POI and the other recognize a ubiquitin protein ligase (E3). In contrast to SMIs, PROTACs suppress POI in a unique mechanism of action (MOA). In brief, once the PROTAC degrader enters the cell, it forms a ternary complex of POIPROTAC-E3. Subsequent ubiquitin-conjugating enzyme (E2)-involved transfer of ubiquitin results in target polyubiquitination and recycle of the PROTAC into the next round of action. The polyubiquitinated POI can be recognized by 26S proteasome and eventually broken into small peptides (Figure 3). Various techniques have been developed to characterize each step of this process [51]. Over 50 proteins have been successfully targeted by this technology, including nuclear receptors, protein kinases, epigenetic regulators, neurodegenerative disease-related proteins, regulatory proteins, anti-apoptotic proteins, virus-related proteins, transcription factors, and even E3 ligases [17, 52]. To date, ARV-110 and ARV-471, two PROTAC degraders targeting AR and ER, respectively, have entered Phase I clinical trials (identifiers: NCT03888612, NCT04072952).

Compared with SMIs, PROTACs possess several unique advantages [16]. Due to the catalytic MOA, PROTACs are intrinsically more potent than SMIs. In addition, the warhead, the pharmacophore that binds the POI, of a PROTAC does not need to bind to the active binding site of the POI to achieve protein degradation. Thus, PROTACs hold the potential of eliciting the desired pharmacological effects by targeting the proteins previously considered to be undruggable. Other advantages include the long-lasting suppression of the POI, an added layer of selectivity to further reduce off-target effect, and ability to target mutated proteins.

## BCL-X_L_-targeting PROTACs

**Figure 4.**
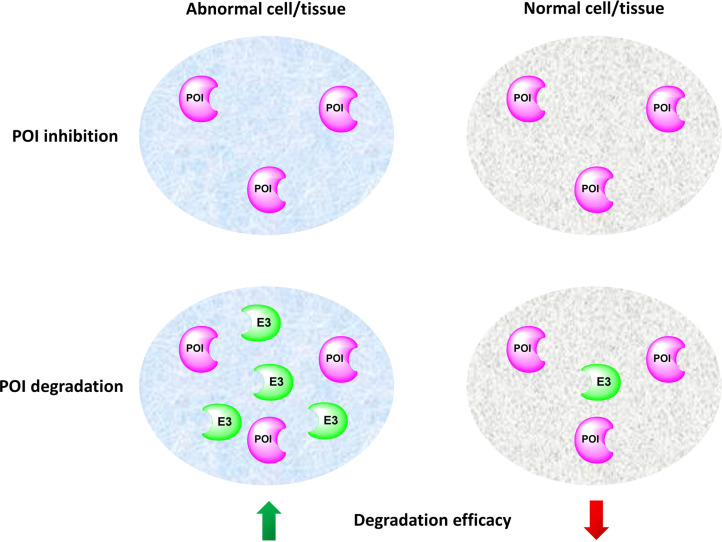
The utilization of PROTAC technology to reduce on-target drug toxicity

Efficacy and safety are two major considerations in selecting a drug candidate. Drug toxicity can be due to off-target or on-target effects. While the off-target toxicity could be addressed by traditional medicinal chemistry approaches, it is extremely challenging to minimize the on-target adverse effect. Besides targeted delivery approaches such as ADC, PROTAC is another potential solution to the on-target toxicity issue. Because PROTACs engage E3 ligases to induce targeted protein degradation, the degradation potency of a PROTAC molecule is also determined by the protein levels of the E3 ligase it recruits. Thus, by recruiting E3 ligases that are relatively higher expressed in the diseased cells/tissues than normal cells/tissues, PROTACs can potentially achieve selective degradation of the POI in the diseased cells/tissues to reduce on-target toxicity (Figure 4).

BCL-X_L_ is an ideal protein for the proof-of-concept study of using PROTAC to overcome the on-target toxicity because thrombocytopenia is known on-target toxicity associated with BCL-X_L_ inhibition in platelets [38, 39]. Our analysis of RNA-sequencing data reveals that all of the commonly used E3 ligases in PROTAC design are expressed at relatively low levels in human platelets [53, 54]. Subsequent western blot assays also suggest that the protein levels of selected E3 ligases along with ubiquitin-activating enzyme (E1) and E2 enzymes are also low in human platelets compared to different tumor and senescent cells [21]. Thus, based on these initial observations, several series of BCL-X_L_-targeting PROTACs have been designed and synthesized by our group [20, 21, 55–57].

**Figure 5.**
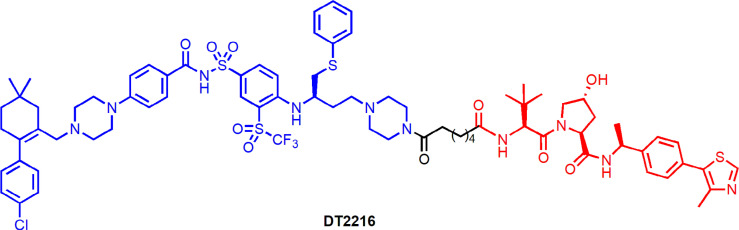
Chemical structure of DT2216 that recruits VHL E3 ligase for BCL-X_L_ degradation

DT2216 (Figure 5) is a von Hippel-Landau (VHL)-recruiting PROTAC with the warhead derived from ABT-263. The solvent-exposed morpholine ring in ABT-263 was replaced by a piperazine ring so that a linker can be attached through an amide bond. DT2216 efficiently induced BCL-X_L_ protein degradation in MOLT-4 T-cell acute lymphoblastic leukemia (T-ALL) cells with a half-maximal degradation concentration (DC_50_) of 63 nM and maximum degradation (D_max_) of 90.8%, and the degradation was dependent on both VHL and proteasome. However, only a small reduction of BCL-X_L_ levels (D_max_, 26%) was observed in human platelets treated with DT2216 at various concentrations (0.037–3.0 μM) [21]. When tested in a cell viability assay, DT2216 was more potent than its parent compound ABT-263 against MOLT-4 cells [half maximal effective concentration (EC_50_) = 52 nM *vs.* 191 nM]. Importantly, DT2216 exhibited no toxicity to human platelets up to 3 μM concentration while ABT-263 was equally toxic to both MOLT-4 cells and platelets (EC_50_ = 237 nM). The reduced platelet toxicity of DT2216 is likely due to a combination of its minimal induction of BCL-X_L_ degradation in platelets, its lower binding affinity to BCL-X_L_ and lower cell permeability than ABT-263. Thus, these results provided strong proof-of-concept evidence that converting BCL-X_L_ inhibitors to PROTACs can significantly reduce the on-target platelet toxicity of the inhibitors.

Interestingly, although DT2216 had a higher binding affinity to BCL-2 than to BCL-X_L_, Bcl-2 degradation was not observed in all cancer cell lines examined. Proteomics studies also showed that DT2216 specifically reduced BCL-X_L_ protein levels [21]. The selective degradation for BCL-X_L_ over BCL-2 was also observed with DT2216 analogs in which the VHL-recruiting moiety of DT2216 was replaced by a celebron (CRBN)-recruiting moiety [55, 56] or inhibitors of apoptosis protein (IAP)-recruiting moiety [57]. It was observed that DT2216 could form stable BCL-2-DT2216-VHL ternary complexes *in vitro* using AlphaLISA assay [58] but not in live cells, as determined by nanoBRET assay [59]. In addition, apparently BCL-2 also lacks a Lys residue in the region where ubiquitin can be transferred to from E2 [21]. These results formed the molecular basis of DT2216 to selectively degrade BCL-X_L_, but not BCL-2. This increased specificity can potentially reduce another on-target toxicity of ABT-263, i.e. neutropenia, because neutrophils depend on BCL-2 for survival [60]. Since BCL-2 inhibition exacerbates chemotherapy-induced neutropenia by suppressing granulopoiesis, while inhibition of BCL-X_L_ can sensitize cancer cells to chemotherapy [44], DT2216 has the potential to be combined with chemotherapy for the treatment of different cancers.

In *in vivo* antitumor efficacy assays [21], weekly administration of DT2216 at 15 mg/kg (ip) was more effective in suppressing MOLT-4 T-ALL xenografts in mice than daily treatment with ABT-263 at 50 mg/kg (po). The combination of DT2216 (15 mg/kg, q7d, ip) and ABT-199 (50 mg/kg, qd, po) induced nearly complete suppression of tumor growth in mice xenografted with NCI-H146 SCLC cells, which depend on both BCL-X_L_ and BCL-2 for survival. In MDA-MB-231 breast cancer xenograft mouse model, DT2216 (15 mg/kg, q4d, ip) in combination with docetaxel (7.5 mg/kg, q14d, iv) was significantly more effective in suppressing the tumor growth than docetaxel alone, indicating that DT2216 can sensitize cancer cells to chemotherapy. In T-ALL PDX models, DT2216 in combination with ABT-199 or chemotherapy (a combination of vincristine 0.15 mg/kg, dexamethasone 5 mg/kg, and L-asparaginase 1, 000 U/kg, ip, q7d) exhibited significantly improved anti-leukemic activity in comparison to ABT-199 or chemotherapy alone. In all these studies, severe thrombocytopenia was not observed and DT2216 was found to be much safer than navitoclax on platelets, indicating that DT2216 is a novel anticancer drug candidate that can more safely target BCL-X_L_.

**Figure 6.**
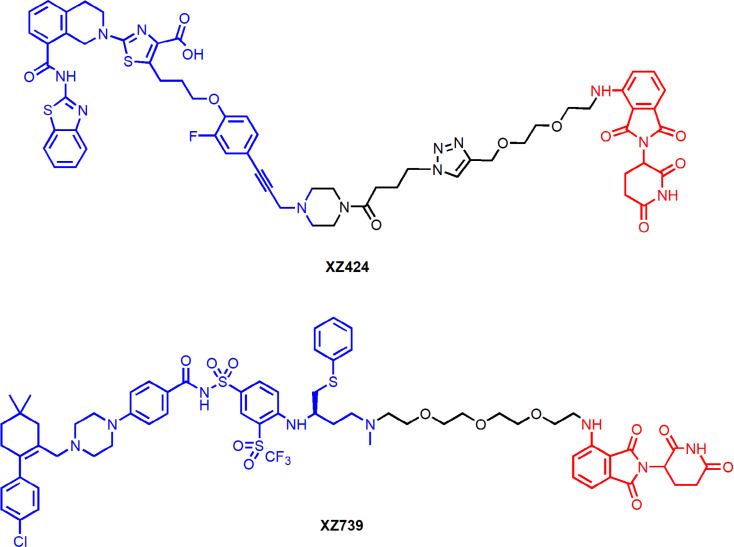
Chemical structures of XZ424 and XZ739 that recruit CRBN E3 ligase for BCL-X_L_ degradation

A-1155463, a potent BCL-X_L_ specific inhibitor, has also been used as the warhead to build CRBN-recruiting PROTACs [20]. CRBN is one of the most frequently E3 ligases used in PROTACs that also shown to lowly expressed in human platelets compared to different tumor cells [20]. The lead compound XZ424 (Figure 6) had similar BCL-X_L_ degradation potency (DC_50_ = 50 nM) and antiproliferative activity (EC_50_ = 51 nM) in MOLT-4 cells when compared with DT2216. XZ424 also showed much lower toxicity to human platelets than its corresponding parent compound A-1155463 (EC_50_ = 1.1 μM *vs.* 7.1 nM). The binding affinity of XZ424 to BCL-X_L_ is similar to A-1155463 [20]. Thus, the reduced platelet toxicity of XZ424 is likely attributable to its minimal induction of BCL-X_L_ degradation and lower cell permeability than A-1155463. Subsequent structural modification led to the discovery of XZ739 (Figure 6), a bioavailable CRBN-recruiting, ABT-263 derived BCL-X_L_ degrader with a DC_50_ of 2.5 nM in MOLT-4 cells [55]. XZ739 is the most potent BCL-X_L_ degrader reported and is 20-fold more potent than ABT-263 against MOLT-4 cells. Notably, DT2216 is less cytotoxic (EC_50_ = 278 nM) in NCI-H146 cells whereas XZ739 displayed strong killing effect (EC_50_ = 25 nM), indicating XZ739, as a single agent, may have the potential to treat tumors that depend on both BCL-X_L_ and BCL-2 for survival. XZ739 also showed good selectivity for MOLT-4 cells over human platelets (> 100-fold).

**Figure 7.**
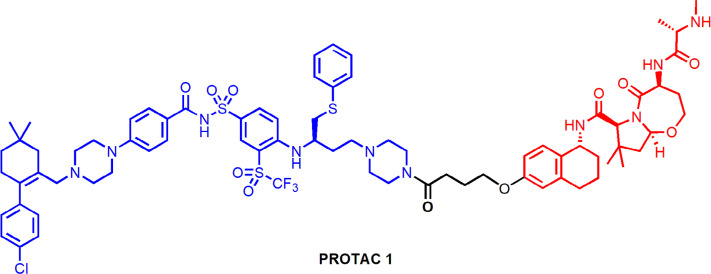
Chemical structure of PROTAC 1 that recruits IAPs for BCL-X_L_ degradation

One of the resistance mechanisms of PROTAC-induced degradation is poor protein expression or mutation of the responsible E3 ligase, which could be addressed by the design of PROTACs that recruit alternative E3 ligases. PROTAC 1 (Figure 7) is a representative degrader in a series of PROTACs that recruit IAP E3 ligases for BCL-X_L_ degradation. It induced BCL-X_L_ degradation and effectively kill malignant MyLa 1929 T-cell lymphoma cells (EC_50_ = 62 nM) [57]. Interestingly, CRBN-recruiting PROTACs including XZ424 and XZ739 showed compromised cytotoxicity in this cell line, likely due to the relatively low CRBN expression. PROTAC 1 appeared to be less toxic to human platelets than ABT-263, which can be attributed to the low XIAP expression in platelets according to Western blot analysis [57]. In addition, PROTAC 1 was able to induce robust BCL-X_L_ degradation across multiple cancer cell lines, suggesting that IAP-based degraders targeting BCL-X_L_ hold the potential of extensive applications in cancer treatment.

**Figure 8.**
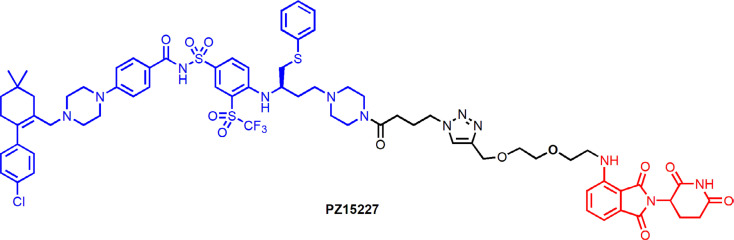
Chemical structure of PZ15227 that recruits CRBN E3 ligase for BCL-X_L_ degradation

BCL-X_L_ PROTAC degraders have also been tested in senescent cells and in relevant animal models. PZ15227, a CRBN-recruiting degrader derived from ABT-263 (Figure 8), selectively induced BCL-X_L_ degradation in senescent cells and non-senescent cells, but not in platelets [56]. Non-senescent cells do not depend on BCL-X_L_ for survival, as a result, PZ15227 selectively induced apoptosis in senescent cells including those derived from WI-38 fibroblasts, IMR90 cells, renal epithelial cells and pre-adipocytes. Overall, compared to ABT263, PZ15227 is much less toxic to human platelets because of low CRBN expression in platelets, but equally or more potent against senescent cells. A galacto-conjugated ABT-263 prodrug, that can be selectively released in senescent cells due to the high levels of β-galactosidase, has been shown to have improved therapeutic window [61]; however, the cellular activity might be compromised because of the decreased cell permeability and incomplete release of the active drug. In contrast, due to the catalytic MOA, PROTACs have the potential to be more potent than the parent compound.

In animal studies, PZ15227 (61 mg/kg, q3d, ip) cleared senescent cells without causing notable thrombocytopenia. Moreover, clearance of senescent cells by PZ15227 improved osteoprogenitor functions. Further, PZ15227 attenuated age-related myeloid skewing and rejuvenated hematopoietic stem cells in naturally aged mice.

## Conclusion

BCL-X_L_ is one of the most important targets for cancer and senolytic therapy. By converting BCL-X_L_ inhibitors to PROTAC degraders, the on-target and dose-limiting thrombocytopenia can be manageable. With further optimization, BCL-X_L_ degraders have the potential to achieve extensive clinical applications. It worth noting that Chung et al. [62], recently reported the first crystal structure of VHL-PROTAC-BCL-X_L_ ternary complex, which could be useful for structure-based design of novel BCL-X_L_ degraders. However, several potential limitations need to be considered during the investigation. PROTACs may have limited efficacy in tissues that have poor expression levels of the E3 ligase they recruit. The acquired resistance to PROTACs has also been reported after chronic treatment with either CRBN- or VHL-based PROTACs [63]. In addition, some CRBN-recruiting PROTACs have the potential of degrading neo-substrates such as C2H2 zinc finger proteins [64]. Further, because PROTACs usually have higher molecular weight compared to SMIs, especially for degraders targeting PPI such as the molecules discussed in this review, most of them have high topological polar surface area (TPSA) and hydrophobicity that is far beyond Lipinski’s rule-of-five. Compared to SMIs, optimization of the pharmaceutical properties of PROTACs could face more challenges. However, it is still not clear whether PROTACs and SMIs should apply the same set of drug-like physicochemical properties. For example, due to the catalytic mode of action of PROTACs, the requirement for cell membrane permeability might be much lower than SMIs.

PROTAC technology enables us to expand the number of proteins which can be targeted for clinical intervention. Utilization of the differentially expressed E3 ligase to overcome the on-target toxicity is one successful example [17, 65]. Similar strategy might be useful for other proteins such as another important anti-apoptotic protein MCL-1. Among five MCL-1 inhibitors that entered clinical trials, two of those were halted due to “safety signal for cardiac toxicity”, which is suspected to be on-target toxicity of MCL-1 inhibition [66]. It will be interesting to investigate if tissue-specific MCL-1 PROTACs can be developed to mitigate cardiac toxicity associated with MCL-1 inhibition.

## Abbreviations

ADC: antibody-drug conjugate
APAF-1: apoptotic protease activating factor-1
BCL-2: B-cell lymphoma 2
BH: BCL-2 homology
CLL: chronic lymphocytic leukemia
CRBN: celeblon
DC_50_: half-maximal degradation concentration 50
D_max_: maximum degradation
E2: ubiquitin-conjugating enzyme
E3: ubiquitin protein ligase
EC_50_: half maximal effective concentration 50
IAP: inhibitors of apoptosis protein
MOA: mechanism of action
MOMP: mitochondrial outer membrane permeabilization
POI: protein of interest
PPI: protein-protein interaction
PROTAC: proteolysis targeting chimera
SCLC: small cell lung cancer
SMI: small molecule inhibitor
T-ALL: T-cell acute lymphoblastic leukemia
VHL: von Hippel-Landau
XIAP: X-linked inhibitor of apoptosis protein

## Declarations

### Copyright

© The Author(s) 2020.
